# Correction: Hsiao et al. Claspin-Dependent and -Independent Chk1 Activation by a Panel of Biological Stresses. *Biomolecules* 2023, *13*, 125

**DOI:** 10.3390/biom13071145

**Published:** 2023-07-18

**Authors:** Hao-Wen Hsiao, Chi-Chun Yang, Hisao Masai

**Affiliations:** 1 Genome Dynamics Project, Department of Basic Medical Sciences, Tokyo Metropolitan Institute of Medical Science, Setagaya-ku, Tokyo 156-8506, Japan; hsiao-hw@igakuken.or.jp (H.-W.H.); yang-cc@igakuken.or.jp (C.-C.Y.); 2 Department of Computational Biology and Medical Sciences, Graduate School of Frontier Sciences, The University of Tokyo, Kashiwa-shi, Chiba 277-8561, Japan

In the original publication [1], there was an error in Figure 1A. Figure 1A,F are the results of fluorescent imaging of γ-H2AX and pChk1, and Figure 1F additionally shows the results of FACS analysis of γ-H2AX and pChk1. We found that the fluorescent images of pChk1 and γ-H2AX in the panels of LPS, hypoxia, and H_2_O_2_-treated cells in Figure 1A were misplaced. We advertently placed the panels identical to those of Figure 1F. Furthermore, Figure 1B in our original article also contained errors [1]. We noticed the control panel in Figure 1B (non-treated Fucci cells stained with γ-H2AX) was misplaced. We mistakenly placed the image of non-treated Fucci cells stained with pChk1(S345) (although there is no apparent difference, since this is a control panel). The corrected Figure 1 appears below.

The authors state that the scientific conclusions are unaffected. This correction was approved by the Academic Editor. The original publication has also been updated.

## Figures and Tables

**Figure 1:**
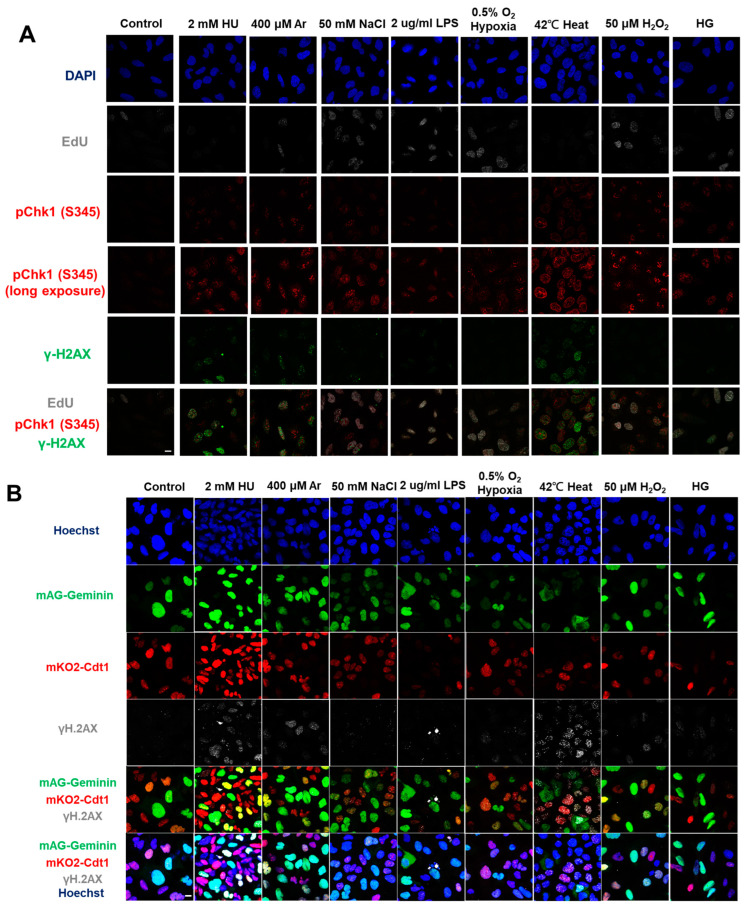

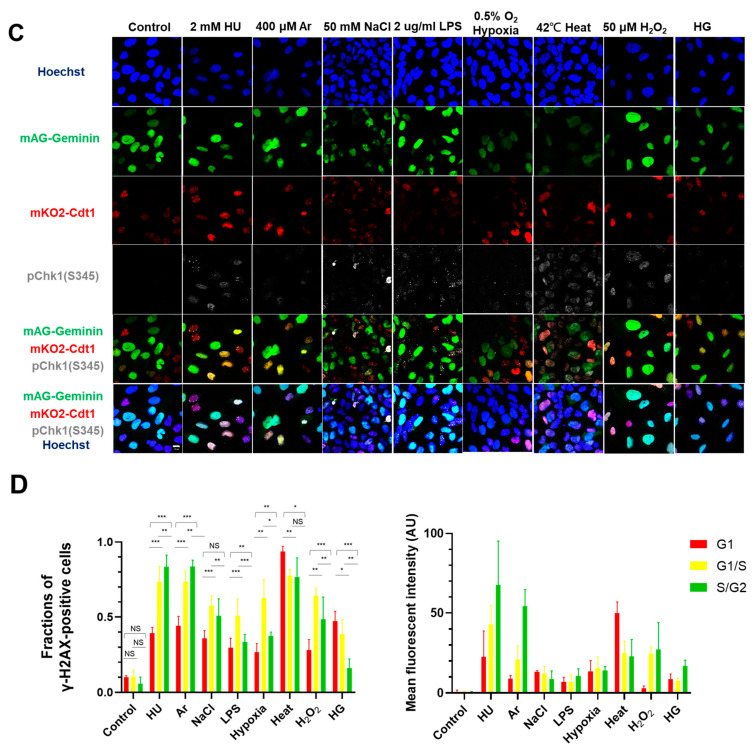

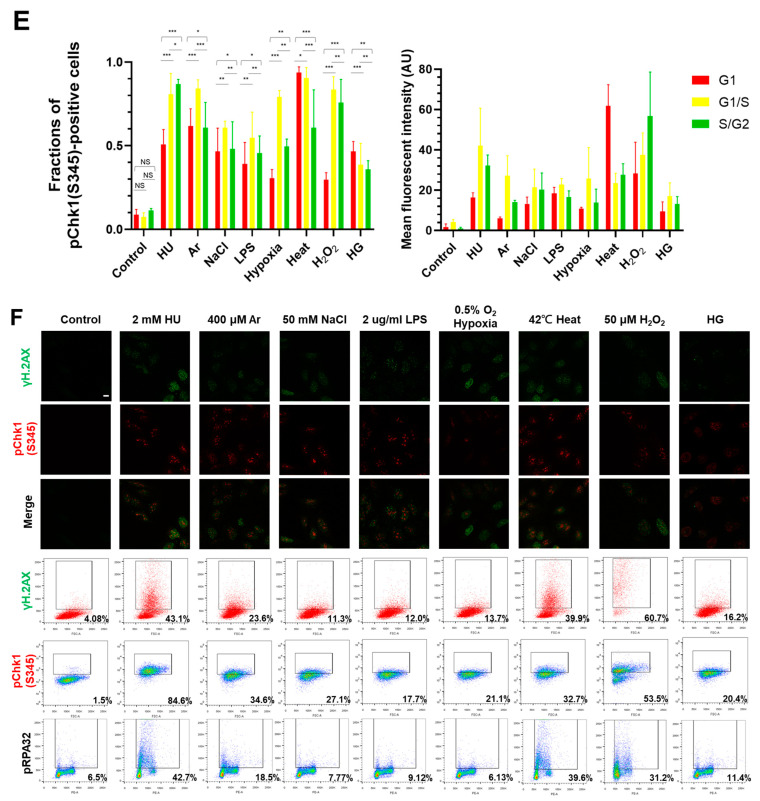

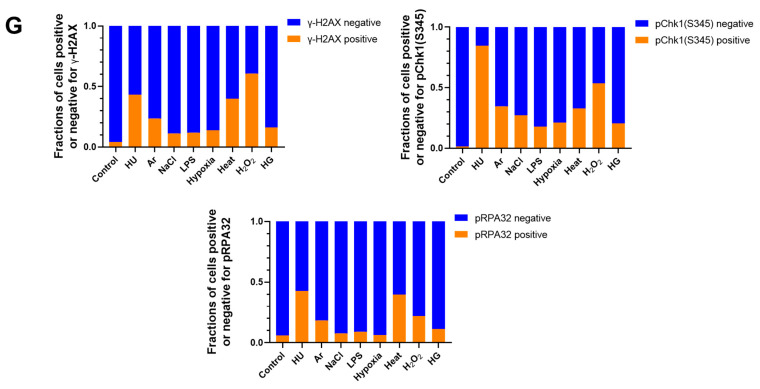
Differential effects on DNA replication and induction of DNA damage and replication checkpoint in a cell-cycle stage-dependent manner by various cellular stresses. (**A**) U2OS cells were exposed to indicated cellular stresses for 3 h, EdU-labeled for 15 min, and stained with indicated markers. Cells were then visualized and analyzed by confocal microscopy (Zeiss LSM780). Representative images are shown. Scale bar is 10 µm. Blue, DAPI (DNA); white, EdU (DNA synthesis); red, pChk1(S345) (replication checkpoint); green, γ-H2AX(DSB). A long-exposed version of pChk1 (S345) is also shown. (**B**,**C**) U2OS Fucci cells were exposed to indicated cellular stresses for 3 h and subjected to immunostaining. Cells were then analyzed by confocal microscopy (Zeiss LSM780). Representative images are shown. Scale bar is 10 μm. Blue, Hoechst (DNA); green, geminin (S/G2 marker): red, Cdt1 (G1 marker); white, γ-H2AX; yellow in the merged image, G1/S boundary. (**D**,**E**) Left: Fractions of U2OS Fucci cells containing γ-H2AX (**D**) and pChk1(S345) (**E**) foci were quantified for each cell-cycle population. Right: The mean fluorescent intensity of γ-H2AX (**D**) and pChk1(S345) (**E**) was quantified for each cell-cycle population. AU: arbitrary unit. (**F**) U2OS cells were exposed to different stresses for 3 h, and then were subjected to γ-H2AX (green), pChk1(S345) (red), and pRPA32 (S4/8) (phosphorylated single-stranded DNA binding protein representing DNA damage) staining, together with flow cytometry analyses. For γ-H2AX (green) and pChk1(S345) (red), cells were observed under confocal microscopy (Zeiss LSM780). Representative data and images are shown. Scale bar is 10 μm. (**G**) Quantification of the data from (**F**,**G**), fractions of γ-H2AX, pChk1(S345), or pRPA32 (S4/8)-positive populations are indicated for cells exposed to various stresses. Statistical analyses in (**D**,**E**) represent the mean values ± SEM under two independent experiments, all of which included three replicates (* *p* < 0.05, ** *p* < 0.01, *** *p* < 0.001, ns: no significant difference).
