# Supplementary material for: Claspin-Dependent and -Independent Chk1 Activation by a Panel of Biological Stresses
Source: Biomolecules. 2023 Jan 7;13(1):125. doi: 10.3390/biom13010125 (PMC9855620; doi:10.3390/biom13010125)
Supplement: Supplementary file 1 [file biomolecules-13-00125-s001.zip › biomolecules-2098521-supplementary.zip.pdf]

## Supplementary Materials

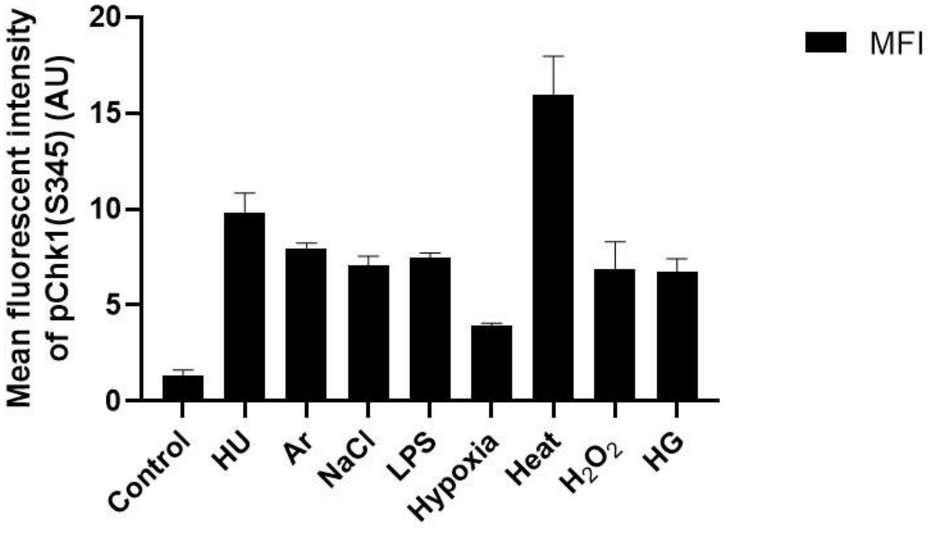


**Figure S1.** Quantification of pChk1 (S345) levels in cells treated with various stresses. The MFI of pChk1(S345) in Figure. 1A was quantified by Image J software. Values are averages of three independent experiments and error bars are included.
